# Developing real-world comparators for clinical trials in chemotherapy-refractory patients with gastric cancer or gastroesophageal junction cancer

**DOI:** 10.1007/s10120-019-01008-9

**Published:** 2019-09-23

**Authors:** Ian Chau, Dung T. Le, Patrick A. Ott, Beata Korytowsky, Hannah Le, T. Kim Le, Ying Zhang, Teresa Sanchez, Gregory A. Maglinte, Melissa Laurie, Pranav Abraham, Dhiren Patel, Tong Shangguan

**Affiliations:** 1 Gastrointestinal and Lymphoma Unit, The Royal Marsden Hospital, Surrey, SM2 5PT UK; 2 Sidney Kimmel Comprehensive Cancer Center At Johns Hopkins, Baltimore, MD USA; 3 Dana-Farber Cancer Institute, Boston, MA USA; 4 Bristol-Myers Squibb Company, Lawrenceville, NJ USA

**Keywords:** Gastric cancer, Gastroesophageal junction cancer, Nivolumab

## Abstract

**Background:**

There are few third-line or later (3L+) treatment options for advanced/metastatic (adv/met) gastric cancer/gastroesophageal junction cancers (GC/GEJC). 3L+ Nivolumab demonstrated encouraging results in Asian patients in the ATTRACTION-2 study compared with placebo (12-month survival, 26% vs 11%), and in Western patients in the single-arm CheckMate 032 study (12-month survival, 44%). This analysis aimed to establish comparator cohorts of US patients receiving routine care in real-world (RW) clinical practice.

**Methods:**

A 2-step matching process generated RW cohorts from Flatiron Health’s oncology database (January 1, 2011–April 30, 2017), for comparison with each trial: (1) clinical trial eligibility criteria were applied; (2) patients were frequency-matched with trial arms for baseline variables significantly associated with survival. Median overall survival (OS) was calculated by Kaplan–Meier analysis from last treatment until death.

**Results:**

Of 742 adv/met GC/GEJC patients with at least 2 prior lines of therapy, matching generated 90 US RW ATTRACTION-2-matched patients (median OS: 3.5 months) versus 163 ATTRACTION-2 placebo patients (median OS: 4.1 months), and 100 US RW CheckMate 032-matched patients (median OS: 2.9 months) versus 42 CheckMate 032 nivolumab-treated patients (median OS: 8.5 months). Baseline characteristics were generally similar between clinical trial arms and RW-matched cohorts.

**Conclusions:**

We successfully developed RW cohorts for comparison with data from clinical trials, with comparable baseline characteristics. Survival in US patients receiving RW care was similar to that seen in Asian patients receiving placebo in ATTRACTION-2; survival with nivolumab in CheckMate 032 appeared favorable compared with US RW clinical practice.

**Electronic supplementary material:**

The online version of this article (10.1007/s10120-019-01008-9) contains supplementary material, which is available to authorized users.

## Introduction

The prevalence of gastric cancer (GC) is generally reported to be decreasing in recent decades [1], but remains the third leading cause of cancer-related mortality worldwide with approximately 783,000 deaths occurring annually [2]. In the United States (US), although prevalence and mortality of GC have fallen over the last 3 decades [1, 3], prognosis remains poor, with a 5-year survival of approximately 31% from 2008–2014 in the US [1]. The most recent report on US cancer statistics by the American Cancer Society, using incidence and mortality data from the National Center for Health Statistics, estimated that GC would account for approximately 27,510 new cases and 11,140 deaths in 2019 [4]. Clinically, management of gastroesophageal junction cancers (GEJC) of the proximal gastric cardia that infiltrate the distal esophagus generally follows guidance for GC [5].

Although GC/GEJC are less common in the US and other Western countries than in Asian countries [6], the burden attributable to this disease in the US remains substantial [7]. When patients with advanced/metastatic (adv/met) GC/GEJC reach the third-line setting, treatment options are limited and clinical outcomes remain poor [8–10]. Promising results have been reported in clinical trials using nivolumab in patients with adv/met GC/GEJC. The ATTRACTION-2 phase III randomized clinical trial (NCT02267343) demonstrated that Asian patients who had received at least 2 prior regimens of systemic chemotherapy for GC/GEJC had improved overall survival (OS) with nivolumab compared with placebo (median OS: 5.3 months vs 4.1 months) [11]. However, it is unclear whether the survival benefit seen with nivolumab in Asian patients compared with placebo also applies to Western/US patients receiving routine clinical care for adv/met GC/GEJC. The CheckMate 032 phase I/II clinical trial (NCT01928394) reported encouraging survival with nivolumab monotherapy in Western patients who received at least 2 prior lines of systemic therapy (median OS: 8.5 months) [12]. However, this clinical trial did not include a control arm, and it is therefore unclear whether nivolumab confers a survival advantage in Western patients receiving routine clinical care.

In recent years, real-world (RW) analyses have increasingly been used to generate external comparator arms for clinical trials [13, 14]. Regulatory bodies have recognized the need for a broader, more flexible framework incorporating RW data for decision-making, as outlined in the 21st Century Cures Act [15, 16]. This study sought to leverage RW data by applying ATTRACTION-2 and CheckMate 032 inclusion criteria and frequency-matching to develop RW comparator arms for these clinical trials, and assessed baseline characteristics, survival outcomes, and duration of therapy (DoT) in RW US patients receiving routine care for adv/met GC/GEJC.

## Methods

### Data source

This retrospective observational study used Flatiron Health’s longitudinal, demographically and geographically diverse database derived from electronic health record (EHR) data from January 1, 2011 through April 30, 2017. The Flatiron database includes data from over 265 cancer clinics (~ 800 sites of care) representing more than 2 million active US cancer patients receiving oncology care in a community practice setting [17], with demographics similar to those of the Surveillance, Epidemiology and End Results (SEER) program [1].

Flatiron data are categorized into disease-specific cohorts for subscription and analysis. The GC/GEJ cohort was used for this analysis. Flatiron patient-level EHR data include structured data (such as demographics, diagnosis codes [International Classification of Diseases, Ninth/Tenth Revisions], visits, laboratory tests, medications, and Eastern Cooperative Oncology Group [ECOG] performance status [PS]) in addition to unstructured data collected via technology-enabled chart abstraction from physicians’ notes and other unstructured documents. Institutional Review Board approval of the study protocol was obtained prior to study conduct and included a waiver of informed consent. Data provided to third parties were de-identified, and provisions were in place for preventing re-identification to protect patients’ confidentiality.

### Study population

**Fig. 1:**
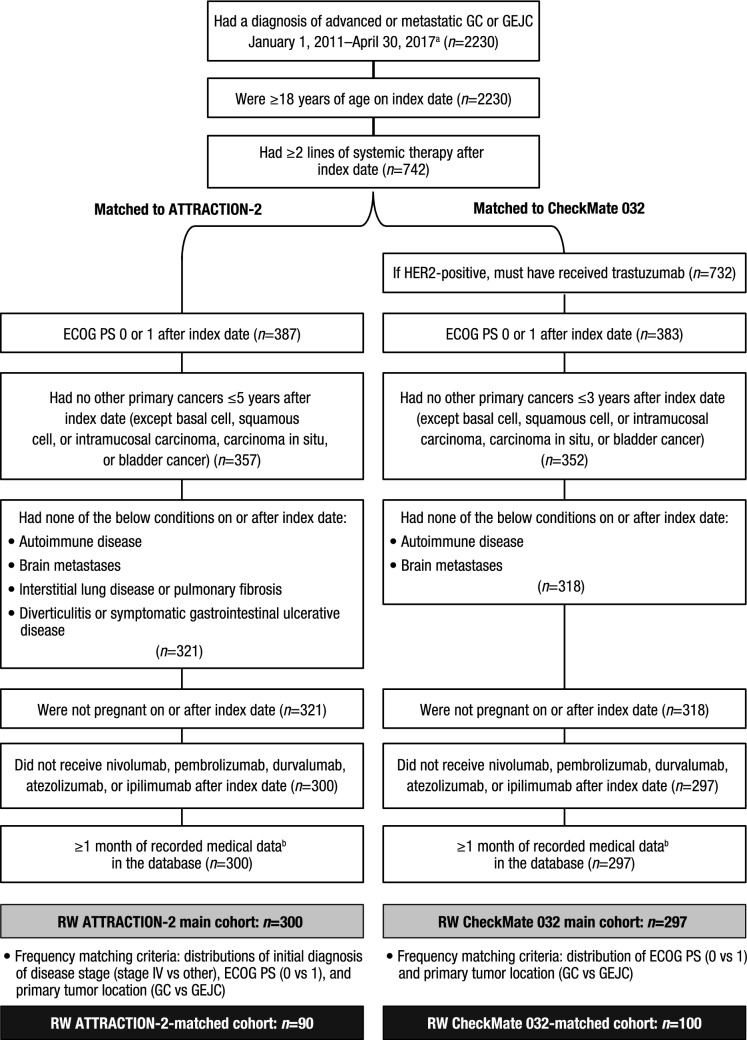
Inclusion/exclusion criteria and patient attrition in RW cohorts matched to ATTRACTION-2 placebo arm and CheckMate 032 nivolumab monotherapy arm. ^a^Excluded patients who were enrolled in clinical trials. ^b^Defined as data from outpatient physician office visits, nonfacility visits, laboratory visits, treatment/procedure visits, or medication administration. *ECOG PS* Eastern Cooperative Oncology Group performance status, *GC* gastric cancer, *GEJC* gastroesophageal junction cancer, *HER2* human epidermal growth factor receptor-2, *RW* real-world

This study involved 2 analyses. One aimed to develop a RW cohort for comparison with patients in the ATTRACTION-2 placebo arm; the second aimed to develop a RW cohort for comparison with patients in the CheckMate 032 nivolumab monotherapy arm (Fig. 1). After identifying a broad group of patients with adv/met GC/GEJC who had received at least 2 systemic treatments after index (the index date was defined as the date of first diagnosis of adv/met GC/GEJC), the RW cohorts for comparison with each trial were developed using a 2-step matching process: first, similar inclusion and exclusion criteria to the ATTRACTION-2 placebo arm (“RW ATTRACTION-2 main cohort”) and the CheckMate 032 nivolumab monotherapy arm (“RW CheckMate 032 main cohort”) were applied. Second, frequency-matching [18] was used to account for differences in population baseline variables that could influence OS, to further align the RW cohorts with the ATTRACTION-2 placebo arm (“RW ATTRACTION-2-matched cohort”) and the CheckMate 032 nivolumab monotherapy arm (“RW CheckMate 032-matched cohort”). Frequency matching is a process used to ensure that the distribution of important characteristics in a population cohort are aligned with those in a comparator cohort. For frequency matching, baseline characteristics in the RW main cohorts that were significantly associated with OS were identified by univariate analysis. Matched cohorts were then generated to ensure these characteristics were represented in the analysis. Eligible patients were subsequently followed up until death, loss to follow-up, or end of study period (last observed visit date up to and including April 30, 2017), whichever occurred first. A subgroup analysis of the RW CheckMate 032 main cohort was conducted, assessing baseline characteristics and survival in patients diagnosed with GC compared with those diagnosed with GEJC.

### Study variables

Baseline variables were recorded for patients in each RW cohort, including demographics (such as age, sex, race, and ethnicity), insurance/practice type, disease stage at initial diagnosis, length of follow-up, ECOG PS, primary tumor location (GC/GEJC), number of prior systemic therapies received, and types of prior systemic therapies. OS was defined as the time from last systemic treatment until death or censoring. Landmark survival rates at 6 and 12 months were defined as probability of survival at 6 and 12 months for each RW cohort. DoT for each line of therapy, defined as time from first to last administration date, was also reported; if DoT was recorded as less than a day, then DoT was considered 1 day. For reference, previously published outcomes data from ATTRACTION-2 and CheckMate 032 have also been included.

### Statistical analysis

Descriptive statistics were used to describe baseline characteristics at index date; frequencies and proportions were used to describe categorical data, and means (standard deviations [SD]), and medians (ranges) were used for continuous data. Median OS was estimated by Kaplan–Meier. Univariate analyses were used to identify baseline variables in the RW cohorts that were significantly associated with OS. Frequency-matching was then used to align these baseline variables in the RW cohorts with those in the clinical trial arms.

## Results

A total of 2230 patients with a diagnosis of adv/met GC/GEJC were identified in Flatiron between January 1, 2011 and April 30, 2017. Of these, 742 patients (33%) received at least 2 lines of systemic therapy after index (Fig. 1) and were eligible to enter the 2-step matching process for each clinical trial comparison.

### RW cohort matched to ATTRACTION-2 placebo arm

After applying similar eligibility criteria to those used in the ATTRACTION-2 trial (step 1), a RW ATTRACTION-2 main cohort of 300 patients was identified; baseline characteristics for this cohort are presented in Online Resource 1. Univariate analyses showed that the primary tumor location (GC/GEJC), initial stage of disease (stage IV/other), and ECOG PS (0/1) were significantly associated with OS in this cohort (Online Resource 2). Frequency-matching on these significant factors resulted in a RW ATTRACTION-2-matched cohort of 90 patients for evaluation with the 163 patients in the ATTRACTION-2 placebo arm (Fig. 1). After frequency-matching, distributions of ECOG PS (ECOG PS 0: 30% vs 29%; ECOG PS 1: 70% vs 71%) and of stage IV disease at diagnosis (50% vs 50%) were similar between the RW ATTRACTION-2-matched cohort and the ATTRACTION-2 placebo arm, respectively (Table 1). The distribution of primary disease site differed between the RW ATTRACTION-2-matched cohort and ATTRACTION-2 placebo arm (GC: 77% vs 83%; GEJC: 23% vs 7%; unknown: 0% vs 10%); owing to underlying differences in US and Asian populations, this characteristic could not be fully matched.

**Table 1:** Baseline characteristics for the RW-matched cohorts and clinical trial arms

	ATTRACTION-2 placebo arm (*n* = 163)	RW ATTRACTION-2-matched^b^ cohort (*n* = 90)	CheckMate 032 nivolumab arm (*n* = 42)	RW CheckMate 032-matched^b^ cohort (*n* = 100)
Age, y^a^				
Mean (SD)	60 (11.9)	66 (12.6)	57 (11.3)	63 (11.8)
Median (range)	61 (26–83)	66 (33–85)	58.5 (29–80)	64 (33–85)
< 65, *n* (%)	95 (58)	41 (46)	32 (76)	52 (52)
≥ 65, *n* (%)	68 (42)	49 (54)	10 (24)	48 (48)
≥ 75, *n* (%)	14 (9)	29 (32)	2 (5)	16 (16)
Male, *n* (%)	119 (73)	61 (68)	31 (74)	73 (73)
Race, *n* (%)				
Asian	163 (100)	7 (8)	0	7 (7)
White	0	46 (51)	39 (93)	55 (55)
Black/African American	0	9 (10)	3 (7)	6 (6)
Other	0	13 (14)	0	22 (22)
Unknown/missing	0	15 (17)	0	10 (10)
Disease stage at diagnosis, *n* (%)				
Stage I and II	21 (13)	15 (17)	2 (5)	12 (12)
Stage III	58 (36)	24 (27)	11 (26)	17 (17)
Stage IV	81 (50)	45 (50)	28 (67)	67 (67)
Unknown	3 (2)	6 (7)	1 (2)	4 (4)
ECOG PS, *n* (%)^c^				
0	47 (29)	27 (30)	20 (48)	47 (47)
1	116 (71)	63 (70)	22 (52)	53 (53)
Primary site of disease, *n* (%)				
Gastric	135 (83)	69 (77)	16 (38)	38 (38)
Gastroesophageal junction	12 (7)	21 (23)	26 (62)	62 (62)
Unknown	16 (10)	0	0	0
No. of systemic treatment regimens received, *n* (%)^d^				
2	29 (18)	50 (56)	18 (43)	60 (60)
3	62 (38)	28 (31)	17 (40)	27 (27)
4+	72 (44)	12 (13)	7 (17)	13 (13)
Regimens received after index, *n* (%)				
Pyrimidine analogues/fluoropyrimidine	163 (100)	85 (94)	42 (100)	94 (94)
Fluorouracil	66 (40)	65 (72)	31 (74)	72 (72)
Capecitabine	68 (42)	35 (39)	20 (48)	38 (38)
S-1 (gimeracil/oteracil potassium/tegafur)	101 (62)	0	1 (2)	0
Taxanes	140 (86)	66 (73)	30 (71)	74 (74)
Docetaxel	52 (32)	28 (31)	21 (50)	28 (28)
Paclitaxel	100 (61)	49 (54)	14 (33)	56 (56)
Paclitaxel albumin	11 (7)	2 (2)	0	1 (1)
Platinum compounds	157 (96)	83 (92)	41 (98)	93 (93)
Carboplatin	2 (1)	27 (30)	12 (29)	38 (38)
Cisplatin	112 (69)	24 (27)	15 (36)	30 (30)
Oxaliplatin	82 (50)	62 (69)	33 (79)	67 (67)
Irinotecan	123 (75)	24 (27)	17 (40)	25 (25)
Ramucirumab	22 (13)	38 (42)	2 (5)	37 (37)
Trastuzumab	22 (13)	9 (10)	9 (21)	15 (15)
Trastuzumab emtansine	2 (1)	0	1 (2)	0

*ECOG PS* Eastern Cooperative Oncology Group performance status, *GC* gastric cancer, *GEJC* gastroesophageal junction cancer, *RW* real-world, *SD* standard deviation

^a^At last systemic treatment in RW cohorts; at study entry in clinical trial arms

^b^Matched on initial diagnosis of disease stage (stage IV vs other), ECOG PS (0 vs 1), and primary site of disease

^c^Flatiron ECOG values obtained within 30 days after last systemic treatment

^d^Any time during observation period in RW cohorts; prior to start of 3L+ therapy in clinical trial arms

**Fig. 2:**
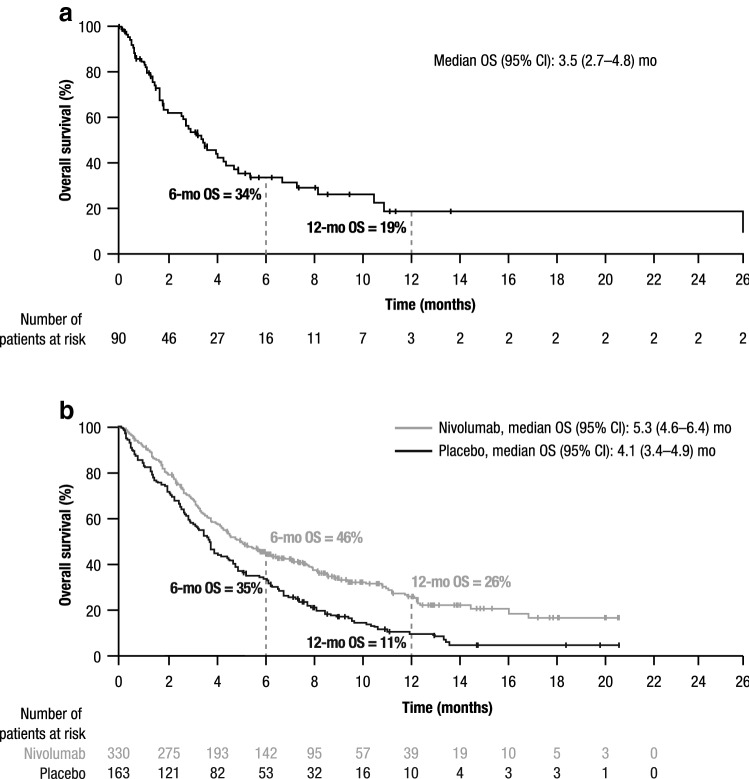
Kaplan–Meier analysis of survival from start of last systemic treatment in **a** RW ATTRACTION-2-matched cohort (*n* = 90) and **b** ATTRACTION-2 placebo (*n* = 163) and nivolumab (*n* = 330) arms. *CI* confidence interval, *mo* month, *OS* overall survival, *RW* real-world. Horizontal axes are matched for comparison purposes; in the RW ATTRACTION-2-matched cohort, 1 patient survived until 42 months

Median OS (95% CI) from start of last systemic treatment in the RW ATTRACTION-2-matched cohort (3.5 [2.7–4.8] months) was broadly comparable with the ATTRACTION-2 placebo arm (4.1 [3.4–4.9] months); median OS (95% CI) in the ATTRACTION-2 nivolumab monotherapy arm was 5.3 (4.6–6.4) months (Fig. 2). Landmark survival rates at 6 and 12 months in the RW ATTRACTION-2-matched cohort were relatively similar to the ATTRACTION-2 placebo arm (6 months: 34% vs 35%, respectively; 12 months: 19% vs 11%, respectively); corresponding landmark survival rates in the ATTRACTION-2 nivolumab arm were 46% and 26% at 6 and 12 months, respectively (Fig. 2).

### RW cohort matched to CheckMate 032 nivolumab monotherapy arm

**Fig. 3:**
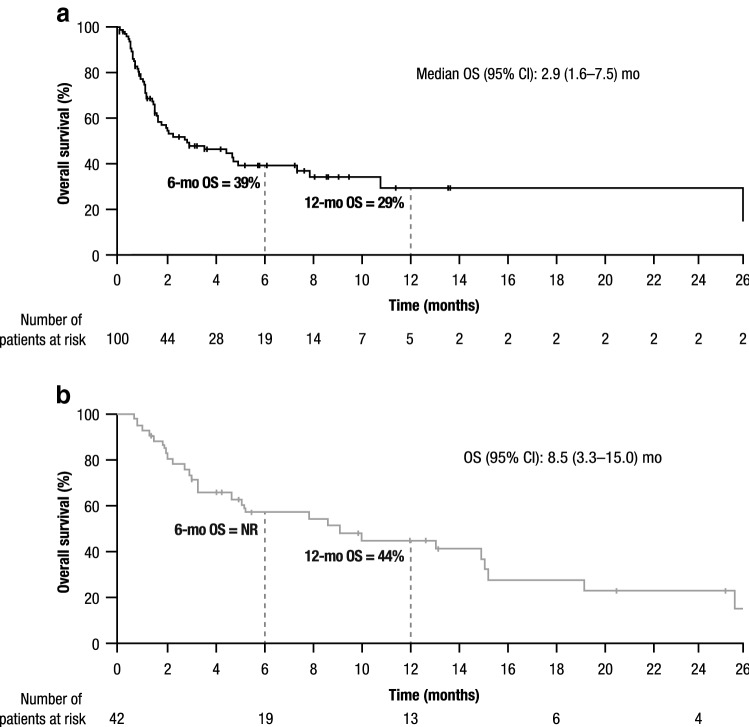
Kaplan–Meier analysis of survival from start of last systemic treatment in **a** RW CheckMate 032-matched cohort (*n* = 100) and **b** CheckMate 032 nivolumab monotherapy arm (*n* = 42). *CI* confidence interval, *mo* month, *NR* not reported, *OS* overall survival, *RW* real-world. Horizontal axes are matched for comparison purposes; in the RW CheckMate 032-matched cohort, 1 patient survived until 42 months

After applying similar eligibility criteria to that used in the CheckMate 032 trial, a RW CheckMate 032 main cohort of 297 patients was identified; baseline characteristics for this cohort are presented in Online Resource 1. Univariate analyses showed that the primary tumor location (GC/GEJC) and ECOG PS (0/1) were significantly associated with OS in the RW CheckMate 032 main cohort (Online Resource 2). Frequency-matching on these factors resulted in a RW CheckMate 032-matched cohort of 100 patients for evaluation with the 42 patients in the CheckMate 032 nivolumab monotherapy arm (Table 1). After frequency-matching, distributions of ECOG PS (ECOG PS 0: 47% vs 48%; ECOG PS 1: 53% vs 52%) and of primary disease site (GC: 38% vs 38%; GEJC: 62% vs 62%) were similar between the RW CheckMate 032-matched cohort and the CheckMate 032 nivolumab monotherapy arm, respectively (Table 1). Median OS (95% CI) from start of last systemic treatment in the RW CheckMate 032-matched cohort was lower (2.9 [1.6–7.5] months) than in the CheckMate 032 nivolumab monotherapy arm (8.5 [3.3–15.0] months) (Fig. 3). Landmark survival in the RW CheckMate 032-matched cohort was 39% and 29% at 6 and 12 months, respectively; 12-month survival in the nivolumab monotherapy arm of CheckMate 032 was 44% (6-month survival was not reported).

When patients in the RW CheckMate 032 main cohort were stratified by tumor location (GC: *n* = 166; GEJC: *n* = 131), the subgroup of patients with GC appeared less likely to be male (58% vs 85%) and younger than 65 years of age (40% vs 52%), and more likely to have an ECOG PS of 0 or 1 (75% vs 58%) than patients with GECJ (Online Resource 3). Median OS (95% CI) was 2.1 (1.8–3.0) months in patients with GC and 1.5 (1.2–2.0) months in patients with GEJC. Landmark survival appeared to be greater in patients with GC compared with patients with GEJC at 6 (30% vs 20%) and 12 months (16% vs 11%).

## Discussion

This “matched” analysis provides US RW context for 2 clinical trials of nivolumab therapy of GC/GEJC. The 2-step matching process enabled generation of RW cohorts that were demonstrably more closely aligned (in terms of baseline characteristics) to patients in the respective clinical trial arms.

Patients receiving nivolumab in ATTRACTION-2 had significantly improved survival (5.3 months) compared with placebo [11]. Survival in patients receiving care in RW US clinical practice (3.5 months) appeared broadly comparable with patients receiving placebo in ATTRACTION-2 (4.1 months) after matching for inclusion criteria, although residual differences in primary disease site persisted after frequency-matching, possibly due to underlying differences between US RW patients and the Asian patients in the placebo arm of ATTRACTION-2. In the matched CheckMate 032 analysis, OS of patients in the RW cohort was 6 months shorter than for patients receiving nivolumab monotherapy in the CheckMate 032 clinical trial [12]. Patients in the RW cohort and CheckMate 032 nivolumab arm appeared to be broadly aligned in terms of baseline demographics.

The CheckMate 032 clinical trial did not include a control arm; the U.S. Food and Drug Administration (FDA) has historically considered drug applications using single-arm trials of therapies only for indications in which no other therapy is available [19]. However, in recent years, the FDA has granted accelerated approval based on surrogate clinical outcomes data from single-arm studies [20–22], complemented by RW studies to generate evidence on safety and effectiveness [23, 24]. RW data can provide information on comparative effectiveness in circumstances in which obtaining equivalent data from randomized controlled trials may be time-consuming and costly [25]. Additionally, clinical trial data may not be representative of the wider population, and may underrepresent clinically important subpopulations. A recent retrospective review of patient records reported that a substantial proportion of patients who failed to meet hypothetical clinical trial eligibility criteria actually received therapy and had similar survival to patients who met the hypothetical eligibility criteria [26].

A previous US-based RW study (assessing data from both the IMS oncology database and Truven MarketScan) reported that approximately 20% of patients who received treatment for advanced GC subsequently received third-line therapy [27]. Given this substantial proportion of adv/met GC/GEJC patients who eventually receive third-line therapy, it is important that efficacious and safe treatments are identified. In this clinical setting, the ATTRACTION-2 and CheckMate 032 clinical trials demonstrate robust efficacy of nivolumab; the present RW analysis suggests that the survival seen with nivolumab in these 2 clinical trials is greater than in matched RW patients in US clinical practice. The potential of programmed cell death ligand 1 inhibitors in this setting is further demonstrated by the results of the KEYNOTE-059 study [28].

To the authors’ knowledge, this is the first study to use clinical trial eligibility criteria and frequency-matching to generate RW cohorts of patients with GC/GEJC. This study is based on a relatively large sample size, and the Flatiron database is generally representative of the wider US oncology community setting [29]. Flatiron demographics are generally similar to those of the SEER program [1]. The Flatiron Health database includes data obtained during treatment at participating clinics and health care providers only, and consequently lacks data related to treatments received at non-participating centers. Additionally, the possibility of selection bias (both in terms of participating centers and in patient entry) may exist, and caution should be taken when applying these findings to broader populations. As with any study assessing records obtained via EHRs, data were collected for disease management rather than for research purposes; consequently, misclassification and incomplete data entry are possible. Lastly, survival comparisons between real-world and clinical trial data must be interpreted with extreme caution; the proportion of patients lost to follow-up is likely to be higher in the real-world analysis, given the close scrutiny and monitoring applied to patients in clinical trials compared with those in real-world clinical practice.

This study used a 2-step matching process to generate 2 RW cohorts with similar baseline characteristics to the ATTRACTION-2 placebo and CheckMate 032 nivolumab monotherapy arms, respectively. These data suggest favorable outcomes with third or later line of nivolumab monotherapy of adv/met GC/GEJC compared with routine clinical care in US patients and with placebo in Asian patients. Notably, Western patients receiving non-immunotherapy as their last line of treatment had broadly similar outcomes to Asian patients receiving placebo, highlighting the relative ineffectiveness of treatments available in US RW practice for advanced lines of therapy. This analysis demonstrates that RW data can be used to generate suitable external control cohorts for clinical trial data lacking a control arm and provide insights on regional applicability of trial data.

## Electronic supplementary material

Below is the link to the electronic supplementary material.

Supplementary file1 (DOCX 22 kb)
