# Correction to: Developing real-world comparators for clinical trials in chemotherapy-refractory patients with gastric cancer or gastroesophageal junction cancer

**DOI:** 10.1007/s10120-019-01021-y

**Published:** 2019-11-03

**Authors:** Ian Chau, Dung T. Le, Patrick A. Ott, Beata Korytowsky, Hannah Le, T. Kim Le, Ying Zhang, Teresa Sanchez, Gregory A. Maglinte, Melissa Laurie, Pranav Abraham, Dhiren Patel, Tong Shangguan

**Affiliations:** 1 Gastrointestinal and Lymphoma Unit, The Royal Marsden Hospital, Surrey, SM2 5PT UK; 2 Sidney Kimmel Comprehensive Cancer Center At Johns Hopkins, Baltimore, MD USA; 3 Dana-Farber Cancer Institute, Boston, MA USA; 4 Bristol-Myers Squibb Company, Lawrenceville, NJ USA

## Correction to: Gastric Cancer 10.1007/s10120-019-01008-9

The article “Developing real-world comparators for clinical trials in chemotherapy-refractory patients with gastric cancer or gastroesophageal junction cancer”, written by Ian Chau, Dung T. Le, Patrick A. Ott, Beata Korytowsky, Hannah Le, T. Kim Le, Ying Zhang, Teresa Sanchez, Gregory A. Maglinte, Melissa Laurie, Pranav Abraham, Dhiren Patel, Tong Shangguan, was originally published electronically on the publisher’s internet portal on 23 September 2019 without open access. With the author(s)’ decision to opt for Open Choice the copyright of the article changed on 25 October 2019 to © The Author(s) 2019 and the article is forthwith distributed under a Creative Commons Attribution 4.0 International License (https://creativecommons.org/licenses/by/4.0/), which permits use, sharing, adaptation, distribution and reproduction in any medium or format, as long as you give appropriate credit to the original author(s) and the source, provide a link to the Creative Commons licence, and indicate if changes were made.

The original article has been corrected.

