# Supplementary material for: Developing real-world comparators for clinical trials in chemotherapy-refractory patients with gastric cancer or gastroesophageal junction cancer
Source: Gastric Cancer. 2019 Sep 23;23(1):133–41. doi: 10.1007/s10120-019-01008-9 (PMC6942583; doi:10.1007/s10120-019-01008-9)
Supplement: Supplementary file 1 — Supplementary file1 (DOCX 22 kb) [file 10120_2019_1008_MOESM1_ESM.docx]

**Developing real-world comparators for clinical trials in chemotherapy-refractory patients with gastric cancer or gastroesophageal junction cancer**

Ian Chau, Dung T. Le, Patrick A. Ott, Beata Korytowsky, Hannah Le, Trong Le, Ying Zhang, Teresa Sanchez, Gregory A. Maglinte, Melissa Laurie, Pranav Abraham, Dhiren Patel, Tong Shangguan

**Journal**: *Gastric Cancer*

**Corresponding author**: Ian Chau, Gastrointestinal and Lymphoma Unit, The Royal Marsden Hospital, SM2 5PT, Surrey, UK

[Ian.Chau@rmh.nhs.uk](mailto:Ian.Chau@rmh.nhs.uk)

**Online Resource 1. Baseline characteristics for the RW main cohorts (prior to frequency-matching to the clinical trial arms)**

|  | **RW ATTRACTION-2 main cohort (*n*=300)** | **RW CheckMate 032 main cohort  (*n*=297)** |
| --- | --- | --- |
| Age, y^a^ |  |  |
| Mean (SD) | 65 (11.8) | 65 (11.7) |
| Median (range) | 66 (33–85) | 66 (33–85) |
| <65, *n* (%) | 133 (44) | 134 (45) |
| ≥65, *n* (%) | 167 (56) | 163 (55) |
| ≥75, *n* (%) | 75 (25) | 72 (24) |
| Male, *n* (%) | 210 (70) | 207 (70) |
| Race, *n* (%) |  |  |
| Asian | 19 (6) | 19 (6) |
| White | 160 (53) | 156 (53) |
| Black/African American | 21 (7) | 23 (8) |
| Other | 46 (15) | 46 (15) |
| Unknown/missing | 54 (18) | 53 (18) |
| Disease stage at diagnosis, *n* (%) |  |  |
| Stage I and II | 22 (7) | 24 (8) |
| Stage III | 51 (17) | 51 (17) |
| Stage IV | 214 (71) | 209 (70) |
| Unknown | 13 (4) | 13 (4) |
| ECOG PS, *n* (%)^b^ |  |  |
| Tested | 274 (91) | 271 (91) |
| 0 | 56 (20) | 56 (21) |
| 1 | 128 (47) | 126 (46) |
| 2 | 60 (22) | 60 (22) |
| 3 | 28 (10) | 28 (10) |
| 4 | 2 (1) | 1 (0.4) |
| Missing | 26 (9) | 26 (9) |
| Primary site of disease, *n* (%) |  |  |
| Gastric | 166 (55) | 166 (56) |
| Gastroesophageal junction | 134 (45) | 131 (44) |
| No. of systemic regimens received, *n* (%) |  |  |
| 2 | 183 (61) | 180 (61) |
| 3 | 80 (27) | 81 (27) |
| 4+ | 37 (12) | 36 (12) |
| Types of prior treatment regimens, *n* (%) |  |  |
| Pyrimidine analogues/fluoropyrimidine | 275 (92) | 272 (92) |
| Fluorouracil | 208 (69) | 205 (69) |
| Capecitabine | 125 (42) | 125 (42) |
| Taxanes | 230 (77) | 227 (76) |
| Docetaxel | 94 (31) | 93 (31) |
| Paclitaxel | 171 (57) | 168 (57) |
| Paclitaxel albumin | 2 (1) | 2 (0.7) |
| Platinum compounds | 274 (91) | 270 (91) |
| Carboplatin | 94 (31) | 92 (31) |
| Cisplatin | 86 (29) | 84 (28) |
| Oxaliplatin | 195 (65) | 191 (64) |
| Irinotecan | 97 (32) | 97 (33) |
| Ramucirumab | 131 (44) | 129 (43) |
| Trastuzumab | 55 (18) | 56 (19) |
| Trastuzumab emtansine | 1 (0.3) | 1 (0.3) |

^a^ At last systemic treatment

^b^Flatiron ECOG values obtained within 30 days after last systemic treatment

*ECOG PS* Eastern Cooperative Oncology Group performance status, *GC* gastric cancer, *GEJC* gastroesophageal junction cancer, *RW* real-world

**Online Resource 2. Univariate hazard ratios derived from univariate Cox regression for selected baseline characteristics**

|  | RW ATTRACTION-2 main cohort | | RW CheckMate 032 main cohort | |
| --- | --- | --- | --- | --- |
|  | HR (95% CI) | *p* value | HR (95% CI) | *p* value |
| Female vs male | 1.14 (0.83–1.55) | 0.421 | 1.14 (0.84–1.56) | 0.407 |
| ≥65 vs <65 y | 0.90 (0.68–1.18) | 0.442 | 0.92 (0.69–1.21) | 0.536 |
| Non-Asian vs Asian | 1.15 (0.47–2.85) | 0.756 | N/A | N/A |
| GC vs GEJC | 0.73 (0.55–0.96) | 0.024 | 0.72 (0.54–0.95) | 0.019 |
| Stage: IV vs other | 1.32 (0.96–1.81) | 0.087 | 1.36 (0.99–1.88) | 0.056 |
| ECOG PS^a^: 2–4 vs 0–1 | 2.62 (1.94–3.54) | <0.001 | 2.55 (1.88–3.44) | <0.001 |
| Line of therapy: 4+ vs 1–3 | 0.99 (0.65–1.50) | 0.954 | 1.03 (0.68–1.56) | 0.896 |

^a^ECOG PS within 30 days of last treatment

*CI* confidence interval, *ECOG PS* Eastern Cooperative Oncology Group performance status, *GC* gastric cancer, *GEJC* gastroesophageal junction cancer, *HR* hazard ratio, *N/A* not applicable, *OS* overall survival, *RW* real-world

**Online Resource 3. Baseline characteristics and clinical outcomes for the GC and GEJC subgroups of RW CheckMate 032 main cohort**

|  | Patients with GC (*n*=166) | Patients with GEJC (*n*=131) |
| --- | --- | --- |
| Age, y^a^ |  |  |
| Mean (SD) | 66 (12.2) | 64 (11.1) |
| Median (range) | 67 (33–85) | 64 (35–85) |
| <65, *n* (%) | 66 (40) | 68 (52) |
| ≥65, *n* (%) | 100 (60) | 63 (48) |
| ≥75, *n* (%) | 46 (28) | 26 (20) |
| Male, *n* (%) | 96 (58) | 111 (85) |
| Race, *n* (%) |  |  |
| Asian | 16 (10) | 3 (2) |
| White | 71 (43) | 85 (65) |
| Black/African American | 20 (12) | 3 (2) |
| Other | 27 (16) | 19 (15) |
| Unknown/missing | 32 (19) | 21 (16) |
| Disease stage at diagnosis, *n* (%) |  |  |
| Stage I and II | 13 (8) | 11 (8) |
| Stage III | 25 (15) | 26 (20) |
| Stage IV | 121 (73) | 88 (67) |
| Unknown | 7 (4) | 6 (5) |
| ECOG PS, *n* (%)^b^ |  |  |
| Tested | 151 (91) | 120 (92) |
| 0 | 35 (23) | 21 (18) |
| 1 | 78 (52) | 48 (40) |
| 2 | 24 (16) | 36 (30) |
| 3 | 14 (9) | 14 (12) |
| 4 | 0 | 1 (1) |
| Missing | 15 (9) | 11 (8) |
| No. of systemic regimens received, *n* (%) |  |  |
| 2 | 107 (64) | 73 (56) |
| 3 | 42 (25) | 39 (30) |
| ≥4 | 17 (10) | 19 (15) |
| Median OS (95% CI) | 2.1 (1.8–3.0) | 1.5 (1.2–2.0) |
| 6-month survival, % | 30 | 20 |
| 12-month survival, % | 16 | 11 |
| Duration of third-line therapy, months (95% CI) | 1.5 (0.0–11.5)  [*n*=59] | 1.8 (0.0–16.8)  [*n*=58] |

^a^ At last systemic treatment

^b^Flatiron ECOG values obtained within 30 days after last systemic treatment

*ECOG PS* Eastern Cooperative Oncology Group performance status, *GC* gastric cancer, *GEJC* gastroesophageal junction cancer, *OS* overall survival, *RW* real-world, *SD* standard deviation
